# Effect of Using Quinoa Flour (*Chenopodium quinoa* Willd.) on the Physicochemical Characteristics of an Extruded Pasta

**DOI:** 10.1155/2021/8813354

**Published:** 2021-04-09

**Authors:** Olga L. Torres, Mariana Lema, Yessica V. Galeano

**Affiliations:** Research Group on Agro-Industrial Sciences, Interdisciplinary Science Institute, Universidad del Quindío, Carrera 15, Calle 12N, Armenia, Quindío, Colombia

## Abstract

Quinoa is a promising raw material for the production of foods with high nutritional quality. This study used quinoa flour (*Chenopodium quinoa* Willd.), egg white, and yucca starch to obtain an extruded pasta. By means of a proximate analysis, the nutritional content of the raw materials, uncooked and cooked pasta, was evaluated. The effects of quinoa flour on the protein composition, physical properties (color, texture, loss through cooking, water absorption, and swelling indices), moisture, DSC, and SEM were evaluated through its comparison with a commercial pasta (control) formulated with quinoa (PCQ). The values obtained during the study were subjected to a simple analysis of variance (ANOVA) to determine the interaction between the factors and the variables by using a statistical program. Incorporation of quinoa flour in the formulations (F1, F2, and F3) increased notoriously the protein content (*p* < 0.05) and decreased the carbohydrate content, and no significant differences were observed for lipids and ash. The energy value increased due to the essential amino acids present in quinoa. The values obtained for *L*∗, *a*∗, and *b*∗ increased with the increase in quinoa flour, and significant differences for *b*∗ (*p* < 0.05) were attributed to the characteristic color of quinoa, drying time, and moisture content. The lack of molecular interaction between starch and protein due to the conditions used in the extrusion process influenced the decrease in rupture strength, increase in the water absorption and swelling indices, and losses due to cooking (8 g/100 g) within an acceptable range. Consequently, affected by the enthalpy of fusion, the starch granules of the quinoa flour did not gelatinize, as observed in the SEM micrographs. The results obtained and the parameters used in the extrusion process influenced the characteristics of the pasta, indicating that quinoa flour is a promising raw material for obtaining gluten-free products.

## 1. Introduction

Production of food pasta may be treated as a mature technological process, considering the acceptance in markets and the generalized use of the end product [1]. It is among the most consumed foods throughout the world due to easy storage, cooking, and versatility when combined with other ingredients. This food is produced by mixing wheat flour or flours from durum wheat and potable water; then, there is a kneading and molding stage and, finally, drying [2]. In addition, pasta is recognized for its high content of complex carbohydrates, low levels of minerals, vitamins, bioactive compounds, and amino acids essential for human beings [3]; thus, various studies have been conducted to improve the protein content of pasta by adding raw materials of plant origin.

Consumers are very demanding with quality requirements and do not easily accept its variability in the product; thereby, it is necessary to address the compositional and functional traits of the raw material [4]. In this respect, the use of quinoa (*Chenopodium quinoa* Willd.) in human nutrition has been recognized by the FAO as an important food crop, whose grains are highly nutritional as a protein source, particularly rich in essential amino acids, like lysine and methionine. In general, quinoa has better nutritional and functional quality compared to cereal grains like corn, oats, wheat, and rice [5] with which doors open for the production of extruded pasta based on quinoa flour. In this sense, the relationship between the conformation of proteins and the characteristics related to the pasta quality can be evaluated in terms of firmness, color, degree of swelling, and loss of solids. Prior studies have evaluated the physicochemical characteristics of pasta formulated with partial substitution of cereals or pseudocereals [6–8], as well as the processing conditions of the end product [2], where the pasta quality is given principally by the complex structural network of gluten in wheat-based products; these characteristics differ from the formation of the protein network that occurs in gluten-free pasta. Until now, few studies have been published relating the protein molecular structure to the texture characteristics of pasta, and those that have been published focus on wheat pasta [4]. Hence, the objective of this project was to obtain extruded pasta through the total substitution of wheat flour by quinoa flour (*Chenopodium quinoa* Willd.) to evaluate its effect on the protein content and physicochemical and optical characteristics.

## 2. Materials and Methods

### 2.1. Raw Material

Quinoa grains, egg white, and commercial yucca starch were obtained at a local market in the city of Armenia (Colombia).

The saponification process was carried out due to saponin compounds located in the pericarp seeds establishing a handicap for quinoa utilization as a food source. Saponins of quinoa are bitter, interfere with quinoa's palatability and digestibility [9, 10], and have been reported to be toxic because of their hemolytic activity and ability to lower the surface tension [11, 12].

### 2.2. Quinoa Saponification Process

The saponification process of the quinoa seeds prior to elaborating the flour was carried out by applying a moist method, which consisted in manually washing with cold water and water at boiling temperature for 60 min. Thereafter, the seeds were placed in aluminum trays at 50°C in a forced air convection oven (Binder, FD-115) for 24 h [13]. After the heat treatment, the seeds were ground in a knife mill (GRINDOMIX GM 200, Retsch) with a 0.5 mm stainless steel sieve to obtain the flour. Finally, the dry flour was kept in a sealed polyethylene bag and stored at room temperature (25°C) [14].

### 2.3. Pasta Elaboration

Three formulations were prepared in the following proportions (*p*/*p*) at levels of 60 g, 50 g, and 40 g/100 g (quinoa flour/total mix) (F1, F2, and F3, respectively), as shown in Table 1.

Each formulation was extruded to homogenize and texturize the raw materials, as proposed by Pardhi et al. [15], using a locally manufactured extruder, equipped with a solid helical screw 80 centimeters long and 3 inches in diameter, powered by 2 Hp 220 V single-phase gear motor and speed variation between 0 and 60 rpm, operated by a PID electronic temperature control with a temperature range from 0 to 200°C. The homogenizing and cooking process for each formulation was conducted at a mean temperature of 60 ± 4.5°C at 60 rpm. In the final section of the product output, spaghetti-type pasta was formed in a formation matrix with 12 openings of 0.8 mm in diameter and a depth of 20 mm each. Thereafter, it was dried in a forced air convection oven (Binder, FD-115) at 70°C until 7% moisture content and stored in sealed bags for later analysis. As a control sample, the study used commercial pasta formulated with quinoa flour (PCQ) acquired in a local market. All analyses were performed in triplicate.

### 2.4. Proximate Analysis

The proximate analysis was evaluated on extruded pasta (uncooked) and on dry extruded pasta (cooked). The parameter determined was crude protein by applying the method by Kjeldhal [16] using a nitrogen-to-protein conversion factor of 6.25. The total carbohydrate level was calculated by subtracting the total fat, protein, ash, and moisture content from 100%, as described by Ayseli et al. [17]. Total fat content was evaluated by using the Soxhlet extraction method [18]. Total ash was determined according to the method in [19]. The energy value was calculated based on the components that produce energy (total protein, fats, and carbohydrates) by multiplying with their specific energy conversion factors [20].

## 3. Moisture Content

The moisture content of the dry extruded pasta was determined by applying the furnace drying method and the general procedures described in [21]. The samples were weighed on an analytical scale (Precisa XB-220A), and the drying conditions were at 105°C for 12 h; then, they were taken to a desiccator for 1 h to cool the sample and weigh it again until obtaining a constant weight.

### 3.1. Physical Properties of the Pasta

#### 3.1.1. Color Measurements

The color of dry extruded pasta was measured in Hunter Lab colorimeter spectrum (Color Quest XE) with D65 illuminant and 10° observer as a reference in the CIEL∗*a*∗*b*∗ space [22]. The results are expressed as *L*∗ (lightness), *a*∗ (redness), and *b*∗ (yellowness). Color change was determined by calculating the color difference index (Δ*E*), as reported by Desai et al. [23].

#### 3.1.2. Texture Characteristics

The mechanical properties of the pasta were evaluated on dry extruded pasta through a flexion and fracture test, a three-point test, to measure the maximum rupture strength (*N*) of the pasta. For its determination, a universal TA-XT2 press was used (Stable Micro Systems, Godalming, Surrey, UK). The three-point test was performed by placing the sample of extruded and dry pasta in the HDP/3PB probe, in such a manner that a sheet moving at a rate of 2 mm/s would break it. This procedure was carried out according to the method described by Mariotti et al. [24]

#### 3.1.3. Losses due to Cooking

The amount of the pasta sample dissolved in the cooking water was evaluated by following the methodology proposed by Desai et al. [23]; this required vacuum filtering, where the filter paper with the moist pasta residue was placed in a forced recirculation furnace at 100°C until reaching a constant weight. The dry pasta residue was weighed, and its real weight (based on Archimedes' principle) was determined and the result was reported as a percentage.

#### 3.1.4. Swelling Index and Water Absorption

The swelling index (SI) and water absorption index (IA) of the dry extruded pasta were determined according to the procedure proposed by Feijoo et al. [25]. For this, 25 g of pasta was taken and subjected to cooking by using the optimal time of 12 min; then, the cooking liquid was drained and left to cool until reaching a temperature of 20°C. Thereafter, the weight of the cooked pasta and dry pasta was registered by using an analytical scale (Precisa XB-220ª). The swelling index (IH) and water absorption index (IA) were calculated through the following formula:

(1) $$\begin{array}{c} \%\text{IH}=\frac{\text{weight of cooked pasta}}{\text{weight of dry pasta}}*100\%, \end{array}$$

(2) $$\begin{array}{c} \%\text{IA}=\frac{\text{weight of cooked pasta}-\text{weight of dry pasta}}{\text{weight of dry pasta}}*100\%. \end{array}$$

### 3.2. Differential Scanning Calorimetry (DSC)

Thermal analysis of the dry extruded pasta was determined using differential scanning calorimetry (NETZSCH 214 Polyma) to measure the starch gelatinization characteristics of the samples by following the methodology described by Lu et al. [26] with some modifications. Milled samples (2.5–3.0 mg) were weighed into an aluminum pan and were wetted with 7 *μ*l of deionized water. The pans were hermetically sealed and allowed to equilibrate 24 h at room temperature. Samples were heated from 10 to 110°C at a rate of 10°C/min. An empty sealed pan was used as a reference. The onset temperature (*T*_o_, °C), peak temperature (*T*_p_, °C), and temperature (*T*_c_, °C) and gelatinization enthalpy (Δ*H*, J/g) were determined using the software provided with the equipment.

### 3.3. Optical Properties

#### 3.3.1. Scanning Electron Microscopy (SEM)

The microstructure of the pasta sample (F1) was evaluated under scanning electron microscopy. This methodology was adapted from that mentioned by Lu et al. [26]. The JSM-6610LV microscope was used (JEOL Ltd., Japan). The sample F1 was systematically observed with increments of 5.000x and 10.000x. Consider the results obtained in the proximate analysis and physical properties of the pasta.

#### 3.3.2. Statistical Analysis

All experiments were performed in triplicate, unless the contrary is indicated. The values obtained during the study were subjected to a simple analysis of variance (ANOVA) to determine the interaction between the factors and variables by using the Statgraphics Centurion XVIII program. Differences were considered significant with *p* < 0.05.

## 4. Results and Discussion

### 4.1. Chemical Composition Analysis

The chemical composition of the uncooked and cooked pasta formulated with quinoa flour is shown in Table 2. The protein value of the three cooked pastas formulated with quinoa flour increased (*p* < 0.05). The results indicated that the protein values in the samples increased by adding quinoa flour, as expected. The high protein content was detected in the F1 formulation. The increase in protein content was influenced by the egg white present in each formulation, but mainly by the quinoa flour, which has a high protein content of about 15% [27]. These results are similar to those by Brunnel et al. [28]. In this sense, quinoa flour is rich in amino acids, such as histidine, lysine, and methionine, besides its starch content between 50% and 60% [29]. In concordance, this property, besides conditioning the pasta behavior, marks a direct relationship between the amount of quinoa flour used in each pasta formulation and the protein content obtained, reaching in the pasta a high content of essential amino acids, as observed in the formulations presented in this research. Conversely, the incorporation of quinoa flour diminished (*p* < 0.05) the carbohydrate content. The extrusion process does not change the protein content [30], but it does influence the decrease in carbohydrate content, due to transglucosidation reactions, which generate atypical glycosidic bonds that are not recognized by amylolytic enzymes [31, 32]. In addition, the amylose present in the formulations is subjected to a conformational order of spiral to helix, making them thermally stable and insoluble; consequently, the amount of carbohydrates present in the formulations decreases [33]. Additionally, no difference (*p* > 0.05) was observed in the lipid content; nevertheless, a decrease in the formulations (F1, F2, and F3) can be attributed to the presence of polar side chains in the fatty acids of the quinoa flour [34]. Additionally, the extrusion process caused the degradation of lipids due to the temperature and screw speed. The ash content associated with the mineral content did not present significant changes.

The lowest energy value (*p* < 0.05) was observed in PCQ in comparison with the three formulations; these energy values may be due to the inclusion of nutrients, such as essential amino acids in quinoa. Desai et al. [23] reported similar results, who added powdered fish rich in polyunsaturated fatty acids and essential amino acids to the pasta, obtaining a higher energy value compared to the control sample. It is also observed that the moisture content of PCQ is significantly higher compared to that of F1 (*p* < 0.05), this is presented as a consequence of the moisture content of the raw material and the temperature used in the extrusion process, and the moisture in F1, F2, F3, and PCQ is within the allowed range where the maximum level is 14%, according to the Codex Stan 249 [35].

### 4.2. Color and Texture Measurements

The color parameter of the cooked pasta is one of the principal quality parameters that consumers consider in a product; in turn, this property is closely related to the raw materials used in its formulation.  Table 3 shows the *L*∗, *a*∗, and *b*∗ coordinates, indicating a mean luminosity in the scale from 0 to 100, besides observing a minor lightness in the control pasta (PCQ) against the samples (F1, F2, and F3). This property of the samples increased as the amount of quinoa flour increased and the percentage of starch provided more whiteness and transparency to the sample. Probably the extrusion contributed to the expansion of the samples with the increasing content of quinoa flour and starch in the formulations, increasing the surface area of the mixtures and favoring light dispersion [36–38]. As a result, it obtained an increase in lightness in F1. Similar values were reported by Guzmán Mora [14]. The trend observed between the values *a*∗ was similar to the values of *b*∗; however, the yellowness (*b*∗) was higher with respect to redness (*a*∗) (*p* < 0.05) attributing these higher values of *b*∗ to the characteristic color of quinoa. In the case of pasta, their color depends on many factors, for example, the carotenoid content of the raw materials and their protein content [39]. The most important factor affecting the color of pasta is the use of plant additives [40], effect of drying time, and moisture content. The Δ*E* values were determined to evaluate the color differences between the PCQ and the pastas (F1, F2, and F3) formulated with quinoa flour, finding that the Δ*E* of the formulated pastas increased when increasing the levels of quinoa flour; similar values were reported by Khan et al. [41]. The Δ*E* values are in the range of 3.0-6.0 and are rated as appreciable and detectable by ordinary people, but they do not make a big difference as indicated in the *Handbook of Color Science* by Yamauchi [42]. Consequently, the extrusion process contributed to the color of the pastas. Likewise, in extruded products, the color is influenced by several factors such as temperature, composition of the raw material, residence time, pressure, shear force, and the temperature at which subsequent drying is carried out.

As with color, the texture parameters, as well as flexion and fracture strength, are important quality characteristics, being a critical point to guarantee product acceptance. Pasta texture is a property controlled principally by a structural network of starch and protein additions of quinoa flour and egg whites. However, firmness is a reflection of the interaction and integrity of the protein matrix present in the pasta after the extrusion process [23].  Table 3 displays the maximum rupture strength (*N*) measurements of F1, F2, F3, and control pasta (PCQ). The values obtained showed that PCQ had the highest firmness of 2.95 N and F1, F2, and F3 had firmness of 2.89, 2.02, and 2.80 N, respectively. Although F1, F2, and F3 had higher protein content, the firmness was lower compared to that of PCQ. This is because there was no interaction between starch/protein, which is why a structural network was not formed. According to Tiga et al. [43], as the quinoa content increases, the gelatinization decreases, and the parameters of temperature, screw speed, and low moisture content in the extrusion process can alter the starch granule and fragment it; as a consequence, there is no interaction with the protein. These results relate to the cooking losses that are presented in Table 4, where it is also observed that F1, F2, and F3 presented the highest values of cooking loss, high swelling, and water absorption index of the pasta; similarly, Foschia et al. [44] published that the higher moisture content and swelling index indicate a lower firmness value of pasta-like products, according to that reported in this research. Similar results in color and maximum rupture strength were reported by Lorusso et al. [45] in pasta substituted partially with fermented quinoa.

### 4.3. Effect of Quinoa Flour on Cooking Loss, Swelling Index, and Water Absorption in Pasta

The cooking quality of pasta is an important factor and is evaluated through the amount of leaching of solids during cooking, absorption of water content, and moisture of the pasta prior to cooking. Table 4 shows the values of the physical properties of the three formulations F1, F2, and F3 and PCQ.

Table 4 indicates that the highest loss through cooking was registered by F1, F2, and F3, while PCQ had a significantly lower (2.53 g/100 g) cooking loss (*p* < 0.05). Feijoo et al. [25] pointed out that the partial or total substitution of wheat flour directly affects upon resistance to disintegration during cooking. In gluten-free pasta, like F1, F2, and F3, the starch polymers are less encapsulated in the matrix due to the lack of a gluten network, thus hindering excessive swelling of the starch granules and, consequently, dispersion of components in the cooking water. The highest value of leaching of solids is seen in F3 (7.98 g/100 g); however, pasta presenting cooking loss < 8 g/100 g is of acceptable quality, according to Foschia et al. [44]. Similar values were reported by Larrosa et al. [46]. Cooking losses are related to interruption in the protein/starch matrix, unequal distribution of water within it, and moisture values of the cooked pasta (61.13 g/100 g) due to the competitive hydration trends [25]. This physicochemical process is related also to the high water absorption index in F1, F2, and F3.

The absorption and swelling indices are a measure of the amount of water absorbed by the pasta during cooking [23]; in this macroscopic event, there is a molecular modification of starch and proteins, principally through hydration. Table 4 shows numerically the influence of the flour quinoa and the processing conditions on the pasta's capacity to absorb water in F1. These swelling index values are similar to those reported by Feijoo et al. [25] and Guzmán Mora [14], presenting values > 90%, a behavior that can be attributed to the abundance of hydrophilic molecules, like amino acids and amylose/amylopectin in quinoa flour, which bind to water molecules. The PCQ showed a significantly lower (*p* < 0.05) swelling index with respect to F1, F2, and F3; this phenomenon is related to the partial substitution of quinoa in its formulation. According to Patiño-Rodríguez et al. [47], this behavior is attributed to the differences in the extrusion process, conditions to formulate the sample, and the circular shape used in the equipment head to shape the pasta. Research conducted by Gallegos-Infante et al. [48] indicated that water absorption is a function of the amylose/amylopectin ratio and the distribution of the length of the amylopectin chain of the food matrix.

### 4.4. Differential Scanning Calorimetry (DSC)

Fusion enthalpy was not found in F1 (extruded and dry); that is, the granules of quinoa starch did not gelatinize. The extrusion process is a processing technique that combines screw rate, temperature, and low moisture content capable of altering the starch granule and fragmenting it by destroying partially or completely the molecule's crystalline structure [49]. This phenomenon is related to the results obtained in the losses due to F1, F2, and F3 cooking, given that when the starch granule is destroyed, there is no interaction with the quinoa protein; hence, there is no strong structural starch/protein network capable of retaining the particles as has been evidenced in the results of the analysis of physical properties, texture, and SEM micrographs.

Similar results were reported by Parada et al. [50] in extruded products. In addition, the PCQ gelatinization temperatures are at 42.7, 64.9, and 70.4°C, corresponding to *T*_o_, *T*_p_, and *T*_c_, respectively, with an enthalpy of -1859 J/g; this last is associated to first-order thermal transitions (crystallization of fat, water, and sugars; starch gelatinization; and protein denaturation). In addition, PCQ in its formulation is composed of corn, rice, and quinoa flour starch, which shows that the content of raw materials and the conditions of the extrusion process affect the interactions between components. A similar behavior was reported by Hernández et al. [32] with a peak temperature of 65.60°C, associated with the highest values of heat absorption, where starch crystallinity occurs and no exothermic peaks were observed.

### 4.5. Scanning Electron Microscopy (SEM)

**Figure 1:**
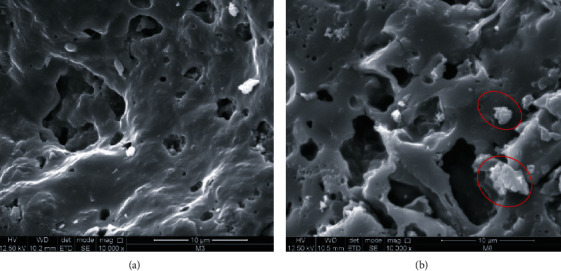
Scanning electron microscopy (SEM) images of PCQ (a) and F1 (b) at 10000x.

The analysis of the scanning electronic microstructure (SEM) of PCQ and F1 (dry extruded pasta) is shown in Figure 1.  Figure 1(a) shows the micrograph corresponding to PCQ, showing a smoother, compact, and homogeneous surface, where the rice starch granules were perfectly embedded in the protein network promoting lower filtration during cooking; therefore, the reason for having fewer cooking losses compared to F1 would be explained. Similar microstructures for rice pasta were reported by Phongthai et al. [7] in their research. On the other hand, the microstructure exhibited in F1 is shown in Figure 1(b); it is evident to have a noncontinuous internal structure with cracks and porous, which, when presented with an open structure, is responsible of the faster absorption of water and percentage of swelling, which could explain the results obtained in the analysis of the loss of cooking. In addition, small starch granules (red circles) are observed to be trapped in the formation of protein networks, which also leads to the possibility of leaching solids during cooking [51].

## 5. Conclusions

The results obtained indicated that the quinoa flour increased the protein content in the formulated pasta. Physicochemical and structural changes were influenced mainly by the conditions used in the extrusion, due to the fact that a molecular interaction of protein with the starch was not formed, a decrease in rupture strength was obtained, and increase in the water absorption index and the swelling power of the pasta formulated in relation to the commercial pasta produces an increase in the leaching of solids and losses due to cooking below 8 g/100 g. Therefore, the raw material and extrusion parameters influenced the pasta; likewise, quinoa is a promising raw material for the preparation of gluten-free products, like pasta, and with new formulations and processing strategies, their functional and nutritional properties could be balanced better.

## Figures and Tables

**Table 1:** Combination of factors yielded by the experimental design.

Ingredients (g/100 g)	Formulations (*p*/*p*)		
	F1	F2	F3
Quinoa flour	60.00	50.00	40.00
Yucca starch	5.00	4.00	3.00
Egg whites	3.22	3.22	3.22
Water	31.78	42.78	53.78

**Table 2:** Chemical composition (g/100 g) and energy value (kcal/100 g) of the components and cooked and uncooked pasta.

Measurements						
Sample	Protein (g/100 g)	Carbohydrate (g/100 g)	Ashes (g/100 g)	Fat (g/100 g)	Energy (kcal/100 g)	Moisture (g/100 g)
Components						
Quinoa flour	13.41 ± 0.27	67.27 ± 0.71	2.53 ± 0.20	5.62 ± 0.27	373.40 ± 2.13	11.14 ± 0.38
Yucca starch	0.48 ± 0.06	88.65 ± 0.27	0.55 ± 0.05	0.47 ± 0.08	360.82 ± 0.83	9.83 ± 0.16
Egg whites	10.16 ± 0.57	1.26 ± 0.73	0.60 ± 0.02	0.23 ± 0.23	47.81 ± 1.07	87.73 ± 0.30

Uncooked pasta						
PCQ	10.51 ± 0.42^a^	80.43 ± 0.47^a^	0.90 ± 0.15^a^	1.18 ± 0.06^a^	374.39 ± 0.94^a^	6.94 ± 0.66^a^
F1	25.56 ± 1.34^b^	67.63 ± 0.54^b^	0.94 ± 0.06^a^	1.96 ± 0.50^a^	390.46 ± 2.36^b^	3.90 ± 0.01^b^
F2	24.23 ± 0.95^c^	69.02 ± 0.44^c^	0.91 ± 0.03^a^	1.94 ± 0.60^a^	390.52 ± 2.01^b^	3.89 ± 0.00^b^
F3	24.78 ± 2.41^c^	68.56 ± 0.44^c^	0.93 ± 0.04^a^	1.93 ± 0.40^a^	390.37 ± 1.87^b^	3.89 ± 0.01^b^

Cooked pasta						
PCQ	10.02 ± 0.04^a^	20.91 ± 0.50^d^	0.89 ± 0.09^a^	0.79 ± 0.09^b^	130.82 ± 2.73^c^	67.73 ± 0.66^c^
F1	19.43 ± 1.03^d^	23.45 ± 1.10^e^	0.91 ± 0.03^a^	1.02 ± 0.04^c^	179.08 ± 0.89^d^	55.89 ± 0.16^d^
F2	18.56 ± 0.56^e^	22.57 ± 1.24^e^	0.88 ± 0.02^a^	1.06 ± 0.06^c^	174.10 ± 5.08^e^	57.22 ± 1.24^e^
F3	17.58 ± 0.63^e^	10.64 ± 1.48^f^	0.90 ± 0.01^a^	1.03 ± 0.04^c^	158.23 ± 4.42^f^	61.13 ± 1.06^f^

PCQ: control sample; F1, F2, F3: pasta prepared with 60 g/100 g of quinoa flour, 50 g/100 g of quinoa flour, and 40 g/100 g of quinoa flour, respectively. Results in the table represent the average of measurements in triplicate. Mean ± standard deviation. Values within a column followed by the same superscript letter are not significantly different from each other (*p* > 0.05).

**Table 3:** Color characteristics (*L*∗, *a*∗, and *b*∗) and texture properties (*N*) of the dry extruded pasta.

Sample	Measurements				
	Color				Rupture strength (*N*)
	*L*∗	*a*∗	*b*∗	Δ*E*	
PCQ	46.80 ± 0.05^a^	2.90 ± 0.10^a^	18.10 ± 0.05^a^		2.95 ± 0.05^a^
F1	51.02 ± 0.00^b^	4.17 ± 0.02^b^	14.11 ± 0.05^b^	5.94 ± 0.11^a^	2.89 ± 0.06^b^
F2	50.11 ± 0.01^b^	3.65 ± 0.01^c^	13.25 ± 0.01^c^	5.45 ± 0.05^b^	2.02 ± 0.07^c^
F3	48.83 ± 0.02^b^	3.70 ± 0.01^c^	12.61 ± 0.03^c^	5.20 ± 0.22^b^	2.80 ± 0.05^c^

PCQ: control sample; F1, F2, F3: pasta prepared with 60 g/100 g of quinoa flour, 50 g/100 g of quinoa flour, and 40 g/100 g of quinoa flour, respectively. Results in the table represent the average of the measurements in triplicate. Mean ± standard deviation. Values within a column followed by the same superscript letter are not significantly different from each other (*p* > 0.05).

**Table 4:** Physical properties of the cooked pasta.

Formulation	Physical properties		
	Losses due to cooking (g/100 g)	Water absorption index (g/100 g)	Swelling index (g/100 g)
PCQ	2.53 ± 0.34^a^	2.78 ± 0.79^a^	95.25 ± 6.26^a^
F1	7.09 ± 1.09^b^	3.35 ± 0.15^b^	101.68 ± 9.89^a^
F2	7.28 ± 0.23^c^	3.15 ± 0.15^b^	101.75 ± 0.62^a^
F3	7.98 ± 0.51^c^	3.56 ± 0.32^c^	99.31 ± 0.58^a^

PCQ: control sample; F1, F2, F3: pasta prepared with 60 g/100 g of quinoa flour, 50 g/100 g of quinoa flour, and 40 g/100 g of quinoa flour, respectively. Results in the table represent the average of the measurements in triplicate. Mean ± standard deviation. Values within a column followed by the same superscript letter are not significantly different from each other (*p* > 0.05).

## Data Availability

For data underlying the findings of the study, researchers may send an email to the authors.
